# Teleconsultation Support for Obstetric Emergencies During the COVID-19 Pandemic in Rural Nepal: Results and Lessons Learned From a Mixed-Methods Study

**DOI:** 10.9745/GHSP-D-23-00423

**Published:** 2025-12-31

**Authors:** Sajana Maharjan, Swaraj Rajbhandari, Liladhar Dhakal, Bhagawati Shrestha, Michaela Hayes, Punya Paudel, Anjana Karki, Binod Dangal, Surya Bhatta

**Affiliations:** a One Heart Worldwide, Lalitpur, Nepal.; b Nidan Hospital, Lalitpur, Nepal.; c One Heart Worldwide, Hoover, Alabama, USA.; d Family Welfare Division, Department of Health Services, Kathmandu, Nepal.; e B & B Hospital, Lalitpur, Nepal.; f Tsho-Rolpa Hospital, Dolakha, Nepal.

## Abstract

In response to the 2020 COVID-19 pandemic, a nongovernmental organization in Nepal piloted a maternal and newborn health helpline program in partnership with the Government of Nepal and the Nepal Society of Obstetricians and Gynaecologists. The program connected service providers in remote areas with real-time clinical support during obstetric and neonatal emergencies via telephone consultations with clinical experts. 551 primary health care facilities across 14 districts of Nepal were included in the program and connected to a roster of 33 clinical expert volunteers from district or tertiary care hospitals. To assess the results of the program, we collected both quantitative and qualitative data, including monthly health facility record reviews from July 2020 to June 2021 and semi-structured interviews with clinical experts and service providers conducted in June 2021. Of the 551 health facilities included in the program, 160 facilities (29%) reported using the helpline during the study period, with 429 teleconsultation cases recorded. We found that 21% of these cases that otherwise would have been referred to a hospital were effectively managed by telephone on-site at rural health facilities, revealing the promising potential for impact using a low-tech solution. Of the health facilities that participated in the program, the helpline was predominantly used in remote facilities where access to higher-level referral centers for emergencies was limited. Feedback from both experts and service providers revealed that the program helped to improve clinical decision-making during emergencies, build confidence and skills of service providers, and improve referral efficiency. Implementation challenges included service providers’ underreporting of helpline utilization, a lack of essential drugs at primary health care facilities, unreliable phone signals, and a lack of financial incentives for clinical experts. The results and implementation experiences shared in this article provide a template for the design and implementation of similar support programs for service providers managing clinical emergencies in rural contexts.

## Abstract (precis)

A helpline program provided real-time clinical support for obstetric emergencies to primary health care service providers through telephone consultation with clinical experts, resulting in benefits in clinical decision-making, timely referral, and case management.

## Key Messages

- A helpline program, designed and implemented during the COVID-19 pandemic, provided real-time clinical support to service providers in remote birthing centers during obstetric and neonatal emergencies via telephone consultation with hospital-based clinical experts.
- Quantitative and qualitative data suggest the program resulted in benefits to clinical decision-making and timely referral for obstetric emergencies while also building the confidence and skills of service providers.
- The program has the potential for scale up and sustainability beyond the pandemic era, especially in remote facilities with limited access to higher-level referral centers, through careful tailoring such as strengthening the pool of dedicated clinical experts and building strong linkages between primary health care facilities and referral centers.
- The study findings will be of interest to governments, policymakers, public health officials, and health workers around the world who can adapt and tailor the modality of remote consultations to their own contexts.

## INTRODUCTION

Nepal has made significant progress in maternal health, reducing maternal mortality by more than a half between 1996 and 2016, from 539 deaths per 100,000 live births to 151 deaths per 100,000 live births.^1^^–^^3^ However, evidence indicates that the positive trajectory may have been severely disrupted by the COVID-19 pandemic,^4^ particularly in underserved rural areas. A nationwide lockdown was implemented from March 24 through June 21, 2020, with partial lockdowns continuing throughout the remainder of the year,^5^ creating significant barriers to maternal and newborn health service delivery and utilization.^6^ A recent study has documented a 50% decline in health facility deliveries and an increase in the institutional neonatal mortality rate from 13 deaths per 1,000 live births to 40 deaths per 1,000 live births over the nine and a half weeks of the initial lockdown.^7^

Similarly, a UNFPA report highlighted an alarming decrease in utilization of reproductive, maternal, and neonatal health services in Nepal’s public health facilities due to widespread fear of COVID-19 transmission, complicated by travel restrictions.^8^ Specifically, travel restrictions severely impacted mobility in rural areas due to preexisting geographical challenges, increasing the need to manage cases locally that were previously referred to higher-level facilities. Anecdotal reports from the government also showed a decrease in institutional deliveries at referral hospitals. This could mean that there was an increase in deliveries at the primary health care center level or in home deliveries. During lockdown, referral to higher-level facilities was difficult, and rural health facilities with only auxiliary nurse-midwives (ANMs) trained in managing normal deliveries would face challenges in dealing with complications. In such situations, connecting ANMs with experts through teleconsultations could provide the ANMs some support to make appropriate clinical case management decisions, which we set out to test.

In rural Nepal, basic maternity services are largely provided by ANMs who receive only 18 months of preservice training after completion of grade 10. Some ANMs have also completed Nepal’s formal skilled birth attendant (SBA) 2-month long certification training. However, these ANMs are trained only to manage normal deliveries, with standard procedures requiring stabilization and prompt referral to a higher level of care for any potential complications.^9^ Limited training and infrequent deliveries leave many ANMs lacking the foundation to maintain their acquired skills and remain clinically qualified. During the COVID-19 lockdown, limited referral options forced these ANMs to manage complicated cases beyond their professional capacity.^10^ A study in Mongolia has shown that in remote geography with huge disparities in quality and access, distance consultation with an expert is effective in reducing maternal and neonatal morbidity and mortality, thus addressing remoteness and rural–urban discrepancy.^11^

In this context, teleconsultations can be an effective approach to provide real-time clinical support to service providers in primary health care facilities by connecting with specialists working in secondary and tertiary hospitals. Studies have shown that the use of telemedicine to link health staff with remote consultations from expert teams has improved diagnosis, management, and patient outcomes, including reductions in maternal and newborn morbidity and mortality.^11^^–^^13^ In addition, telehealth also improved the quality of care by facilitating timely referral to higher center.^11^ Late referral due to lack of knowledge about obstetric emergency warning signs can be lethal for both mother and fetus.^14^ To bridge these gaps in service delivery, the nongovernmental organization One Heart Worldwide and the Nepal Society of Obstetricians and Gynaecologists piloted a maternal and newborn health (MNH) helpline program to connect isolated service providers with real-time clinical support during obstetric and neonatal emergencies via telephone consultations with clinical experts. The program team at One Heart Worldwide implemented the program while the research team conducted this study.

This article aims to describe the design, implementation process, and lessons learned from the implementation of the MNH helpline program for obstetric emergencies during the COVID-19 pandemic in rural Nepal. The study is based on a review of health facility records from July 2020 to June 2021, in-depth interviews of clinical experts and service providers, and surveys of participating service providers.

## PROGRAM DESCRIPTION

Developed in collaboration with the Government of Nepal and the Nepal Society of Obstetricians and Gynaecologists, the MNH helpline was created to assist rural MNH service providers in clinical decision-making during the COVID-19 pandemic. It was designed in anticipation of obstetric or neonatal emergencies requiring additional guidance from specialists or cases where immediate transfer might normally be necessary but not possible due to the COVID-19 lockdown or other adverse conditions.

The program was rolled out in 551 primary health care (PHC) facilities, which includes health posts and primary health care centers, providing MNH service delivery in 14 districts of Nepal, specifically Bhojpur, Dhading, Dolakha, Ilam, Kavrepalanchok, Khotang, Nuwakot, Okhaldhunga, Ramechhap, Sankhuwasabha, Sindupalchok, Solukhumbu, Taplejung, and Udayapur. All these facilities conducted deliveries and had newborn corners but did not have caesarean delivery services. In Nepal, these PHC facilities provide PHC services only, and they refer to secondary and tertiary health care facilities. At the district level, secondary health care services, including caesarean delivery services, are provided mainly by district hospitals.

A roster was created of 33 clinical expert volunteers including obstetricians, general practitioners, and medical officers with advanced SBA training, based in district or tertiary care hospitals. Service providers at each of the 551 PHC facilities then received the names and phone numbers of 3 to 5 experts from corresponding district-based staff of the implementing organization. In-person or virtual orientation sessions on the MNH helpline program was provided to service providers, depending on current travel permissions. During orientation, the service providers were instructed to prioritize calling experts from their corresponding district when they needed support. If the call failed to connect, they could then call other experts from tertiary hospitals on the roster.

A secondary objective of the program was to strengthen the referral system and to build the network between referral centers and PHC facilities. Therefore, service providers also received a printed poster containing contact details of local transport/ambulance drivers, referral centers, and other relevant stakeholders from their municipalities, such as the municipal health officials and the Ward Chairperson (a locally elected representative), for support. The service providers were also oriented on airlifting procedures and referral procedures for making referrals, which included filling referral slips, arranging transport, and informing the referral center about the referred case. In Nepal, there is a free airlifting program in selected remote districts through which the government provides free helicopter service to transfer women to referral centers under the recommendation of health workers and the district chief.

The service providers were provided phone credit worth 200 Nepalese Rupees (about US$2) per month to compensate for the cost of the phone calls and were instructed to maintain a record of all calls in telephone logbooks, including the date and time of each call, the name of the expert contacted, the reason for the call, the consultation received, the actions performed, and the outcome of the case. If the case was referred to a higher-level facility, the means of transportation used for the referral and the outcome of the referred case were also recorded.

## METHODS

### Study Design

This was a mixed-method study using both quantitative and qualitative data. Quantitative data on the use of the MNH helpline were collected prospectively on a monthly basis from July 2020 to June 2021. The data collected included characteristics on the use of the helpline, such as number and type of cases consulted, reasons of consultation, outcome of consulted cases, and means of transportation used to reach the referral center. For the qualitative data, telephone interviews were conducted with both service providers and the clinical experts in June 2021 to uncover the perceived benefits and usefulness of helpline, reasons for not using the helpline, and challenges faced by experts in providing consultation. We did not triangulate the data since the objectives of the quantitative and qualitative methods differed.

### Study Settings

The study was carried out in all the PHC facilities providing delivery services in the 14 districts named earlier.

### Study Population

Data collection was done at both the health facility and individual levels. At the health facility level, all 551 PHC facilities providing labor and delivery services in the 14 districts were included in the study.

At the individual level, all 33 participating clinical experts were initially selected for interview. Of these, 28 were available for interview and included in the study. To explore the reasons for not using the helpline and the perceived benefits and challenges, the service providers were grouped into 3 categories:

1. Never used MNH helpline (14 interviews)
2. Used MNH helpline only once (14 interviews)
3. Used MNH helpline 2 times or more (14 interviews)

From each group, 1 service provider per district was selected for interview, resulting in a total of 42 interviews. Service providers from the PHC facilities who had received mobile phone top-ups were selected for interviews. The reason for not using the helpline was asked to category one service providers who had never used the helpline. Those using the helpline only once might have had different experiences than those using the helpline 2 or more times. Thus, these service providers were grouped into separate categories to explore the perceived benefits and challenges of using the helpline.

### Data Collection and Analysis

#### Quantitative data collection at the health facility level

Telephone and vehicle logbooks were provided to health facilities in which the service providers recorded the details of the cases for which they used the MNH helpline. Services providers then sent the photographs of telephone and vehicle logbooks to district teams using Viber or Messenger on a monthly basis. District teams entered the received details in a designated Google Sheet. The research team then analyzed the data based on data filled in the Google Sheet.

Telephone logbooks included the date and time of the call, name of the clinical expert, the reason for the call (antenatal, delivery, postnatal, and newborn care), advice received from the expert, actions taken after receiving the advice, and outcome of the case (successfully managed at the health facility or referred to the next level of care). In the vehicle logbook, the mode of transportation used by the referred case (ambulance/hired vehicle/stretcher/helicopter) and the cost incurred for transport from a PHC facility to a higher-level referral center were also recorded.

#### Qualitative data collection at the individual level

Research assistants conducted telephone interviews with 28 clinical experts and 42 service providers at PHC facilities using an interview guide. For clinical experts, the interview guide included questions on their experiences providing consultations, perceptions on usefulness of the program, and challenges using the helpline. For service providers, the interview guide covered their experiences, perceptions, challenges, and recommendations to improve the helpline. Those who did not use the helpline were only asked about their reasons for not using the helpline.

#### Data analysis

For the quantitative data, we performed descriptive analysis by calculating frequencies and percentages using SPSS. For the qualitative data, we conducted thematic analysis. The open-ended responses were read thoroughly and then coded. Codes were grouped into appropriate themes.

### Ethics

Ethical approval was obtained from the Nepal Health Research Council (registration number 437/2020).

## RESULTS

### Use of the MNH Helpline

Of the 551 health facilities where the MNH helpline program was implemented, only 160 facilities (29%) reported using the MNH helpline between July 2020 and June 2021. From these 160 facilities, 429 cases of consultations with clinical experts were reported. The helpline was used more during the initial phase of the program and in the monsoon season (June to September) (Figure). Table 1 shows large variation in helpline use between districts. Facilities in Dolakha, Dhading, and Kavre reported using the helpline for 50 cases or more whereas in Illam the helpline was used for only 4 cases. In total 1,523,712 Nepalese Rupees (approximately US$1,500) was spent on mobile top-up for service providers.

**FIGURE:**
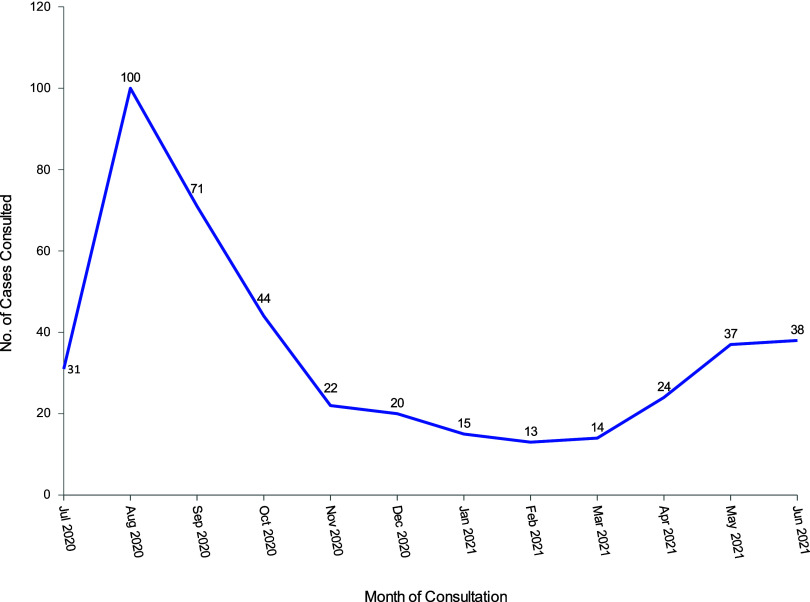
Number of Teleconsultations Reported by Month, 14 Districts of Nepal, July 2020–June 2021 (N=429)

**TABLE 1.** Number of Teleconsultations Reported by District, 14 Districts of Nepal, July 2020–June 2021 (N=429)

**District**	**2020**						**2021**						**Total**
	**Jul**	**Aug**	**Sep**	**Oct**	**Nov**	**Dec**	**Jan**	**Feb**	**Mar**	**Apr**	**May**	**June**	
Bhojpur		3	1	1	1								**6**
Dhading	7	22	14	7	3	3							**56**
Dolakha	2	3	11	3	3	1	4	2	4	4	14	14	**65**
Ilam		2		2									**4**
Kavre	5	10	5	3	2	2		4	3	2	9	5	**50**
Khotang		3	3			2	1	1		1	1	2	**14**
Nuwakot	3	12	4	1								2	**22**
Okhaldhunga	2	2	4	6	1	1		2		2	1	3	**24**
Ramechhap	2	9	5	4	1	2	3	2	1	1	2	1	**33**
Sankhuwasabha	4	8	5	6	4	6	2		1				**36**
Sindupalchowk	3	11	8	1	1	1				10	5	7	**47**
Solukhumbu	1	5	1	1	2						2		**12**
Taplejung		3	5	8	3	2	2		2	1		1	**27**
Udayapur	2	7	5	1	1		3	2	3	3	3	3	**33**
**Total**	**31**	**100**	**71**	**44**	**22**	**20**	**15**	**13**	**14**	**24**	**37**	**38**	**429**

### Characteristics of the Consultation Cases

Regarding the time of consultation, 39% of the cases were consulted during the night (between 8 pm to 8 am) while 61% of cases were consulted during the day (between 8 am to 8 pm) (Table 2). More than half (56.4%) of the teleconsultations concerned delivery-related problems, followed by antenatal (28.0%) and newborn care (12.8%). More than half of the cases (53.7%) were consultations with clinical experts from within the same district. The top 10 complications prompting consultations with experts were prolonged labor, eclampsia, post-dated pregnancy, retained placenta, fetal malpresentation, leaking of amniotic fluid, postpartum hemorrhage, antepartum hemorrhage, irregular fetal heart sound, and Rh incompatibility, as detailed in Table 2. There were also 8 cases of abortion and 3 cases of COVID-19. All the consulted cases were related to maternal and newborn health issues except 1 case of insect bite.

**TABLE 2.** Characteristics of the Reported Teleconsultations, 14 Districts of Nepal, July 2020–June 2021 (N=429)

**Categories**	**No. (%)**
**Affiliation of experts providing consultation**	
Within the district	243 (53.7)
Outside the district	186 (43.3)
**Type of cases consulted**	
Antenatal	120 (28.0)
Delivery	242 (56.4)
Postnatal	10 (2.3)
Newborn	55 (12.8)
Gynecology	2 (0.4)
**Time of consultation**	
Day (8 am to 8 pm)	263 (61.0)
Night (8 pm to 8 am)	166 (39.0)
**Reasons for consultation**	
Prolonged labor	126 (29.0)
Eclampsia	31 (7.2)
Postdated pregnancy	20 (4.6)
Retained placenta	17 (3.9)
Fetal malpresentation during delivery (breech/transverse)	14 (3.2)
Per vaginal leaking	14 (3.2)
Postpartum hemorrhage	13 (3.0)
Antepartum hemorrhage	12 (2.8)
Irregular/absent fetal heart sound	9 (2.1)
Rh incompatibility	9 (2.1)
Abortion	8 (1.8)
COVID-19 positive	3 (0.7)
Other	153 (35.0)
**Outcome of the cases**	
Managed inside PHC facilities	90 (21.0)
Referred and managed	311 (72.5)
Neonatal death	24 (5.6)
Stillbirth	3 (0.7)
Intrauterine fetal death	1 (0.2)
**Means of transportation used to reach referral center**	
Ambulance	128 (29.8)
Hired vehicle	127 (29.6)
Public vehicle	36 (8.4)
Helicopter	20 (4.7)
Stretcher and truck	2 (1.4)

### Outcome of the Consultation Cases

Of the 429 consultation cases, just over one-fifth (21%) of the cases were managed successfully at the PHC facility with the support of consultation with experts, which would have otherwise been referred to a higher level of care. The majority of the cases (72%) were referred, and about 6% resulted in neonatal deaths and stillbirths.

### Means of Transport Used to Reach Referral Center

Nearly one-third (29.8%) of referred patients used an ambulance and a similar percentage (29.6%) used a hired vehicle, whereas 4.7% were evacuated by helicopter and the remaining used public vehicles, stretchers, and trucks for transport. The average cost spent for transportation to reach the referral center was 5118 Nepalese Rupees (about US$50) (range, 500 to 20,000 Nepalese Rupees, or US$5 to $20).

### Details of the Cases Evacuated by Helicopter

Of the 20 cases evacuated by helicopter, 3 were for eclampsia, 3 for postpartum hemorrhage, 3 for retained placenta, 3 for prolonged labor, and 1 each for premature rupture of membrane, preterm birth, preterm labor, sepsis, fetal distress (meconium stained liquor), triplet delivery, post-dated pregnancy, and malpresentation with hand prolapse. Among the helicopter-lifted cases, all mothers survived but 2 neonatal deaths occurred (due to meconium aspiration syndrome and preterm delivery).

### Perceived Usefulness of the MNH Helpline

Both service providers and clinical experts found the helpline program useful, especially in remote areas far from referral centers and lacking proper referral systems. Those interviewed commented on usefulness of the helpline program primarily for:

- Helping with clinical decision-making during emergencies, thus contributing to saving lives of mothers and newborns
- Managing cases inside PHC facilities that would otherwise have been referred
- Timely referral and avoiding delays
- Building confidence and skills of service providers working in PHC facilities

#### Helping With Clinical Decision-Making During Emergencies

The majority of the service providers interviewed reported that the MNH helpline program had been helpful for their clinical decision-making during complicated cases, thus helping save the lives of mothers and newborns. Service providers explained that they had limited knowledge and skills, and when they encountered complications, they were confused and afraid. Through the helpline, they were able to communicate directly with clinical experts and make proper decisions in a timely manner.

*Doctor’s suggestions are always helpful for us, it’s easy for taking decision after talking with them. –* Service provider

*In one case, the mother had a high fever and blood pressure. After a video call consultation with a doctor, the patient received an injection and had a normal delivery. Thus, the helpline has helped in saving the life of a mother. –* Service provider

#### Managing Cases Inside PHC Facilities, Saving Patients Money

Most of the experts interviewed reported that, as a result of advice provided through the helpline system, many cases were managed inside health facilities, without the need for a referral. Some clinical experts reported they had even used video consultation to manage cases.

Service providers also reported that they were able to manage cases through careful follow-up of instructions provided by the expert they had consulted and were therefore able to avoid unnecessary referrals, saving patients’ time and money.

*Due to my consultation, two cases of PPH were managed inside the health facility. –* Clinical expert

*Breech delivery case was there, I provided video consultation and baby was normally delivered. –* Clinical expert

*There was one case with no progress of labor, it was late night and there was no vehicle to refer. Using helpline, I consulted with the clinical expert and gave oxytocin as per his instruction, then a healthy baby was normally delivered. –* Service provider

*Once, the doctor received a call at night around 1 am and asked the fetal heart rate of the baby and said don’t be afraid, thus we didn’t refer and baby is fine now. This program has helped in avoiding unnecessary referral and saved the patient’s money. –* Service provider

#### Timely Referral and Avoiding Delays

Service providers reported that the helpline has aided in early decision on referral, which avoided unnecessary delays. They further shared that it becomes easy to convince a patient’s family that they have consulted with an expert who suggested early referral.

Several clinical experts shared that referral can be expedited due to the helpline. Usually, the first referral center is the district hospital and, if the case cannot be managed there, it will again be referred to another higher-level facility. Using the helpline, service providers directly consulted with the clinical expert at the district hospital and if the case could not be managed there, it was directly referred to a tertiary hospital, avoiding delays resulting from referral from one hospital to another. In addition, clinical experts shared that the helpline helped in expediting the airlifting process. It used to take more than 12 hours to airlift a patient but, in part due to these helpline links, the health facilities were able to complete the airlifting within 4 hours.

*There are some cases which cannot be managed at the District Hospital. In that case, we ask service providers to directly refer the patients to Kathmandu. In such situations it avoids delays and save time.–* Clinical expert of district hospital

#### Building Confidence and Skills

Many of the clinical experts interviewed commented that service providers working in PHC facilities had limited knowledge and skills. Consulting with clinical experts during emergencies provided them an opportunity to learn and practice, thus building their self-confidence, knowledge, and skills. A number of service providers also reported that consulting with experts had increased their confidence and skills.

*Sometimes even in normal cases, service providers say that I will refer, but I asked them I am with you, please go ahead and they perform and then the case is solved. Due to lack of skill and confidence, service providers prefer referring. But after my consultation, the case is managed inside the health facility. This will ultimately help in building service provider confidence and skills. –* Clinical expert

### Challenges Faced by Clinical Experts

Clinical experts reported 4 major challenges while providing consultations:

- Lack of competencies of service providers to manage cases per instructions provided by the expert
- Non-availability of lifesaving drugs and medicines for managing complicated cases
- Lack of consultation incentives
- Disruptions to family and personal life, especially when receiving calls at night

#### Lack of Competencies of Service Providers

Clinical experts commented that service providers working in remote areas have limited knowledge and skills, hindering their ability to fully follow directions provided during their consultations.

*I asked to give a magnesium sulfate injection, but the nursing staff was not confident giving the injection. – Clinical expert*

*When I asked the nurse about the cervix dilatation, she reported it was 7 cm or 4 cm. After referral, on examination we found no dilatation. Service providers do not understand what we say due to their limited knowledge. – Clinical expert*

#### Non-availability of Lifesaving Drugs

Most of the clinical experts mentioned non-availability of drugs as one of the biggest challenges they faced while providing consultation, resulting in the inability of service providers to follow through on directions given.

*Some lifesaving drugs cost only 15 cents, but even those were not available at the health facility. It made me very sad. – Clinical expert*

#### Lack of Consultation Incentives

Some experts explained that this is volunteer work and that many times they are busy driving or performing operations so they cannot receive calls, and they do not feel obliged to call back. Thus, they suggested the need for incentives to motivate clinical experts.

*If there were some incentives, we would be more motivated to provide consultation. It involves a significant amount of our time. – Clinical expert*

#### Disruptions to Personal Life

Three experts said that the calls disturbed their family and personal life, since they received calls at odd hours. Two experts added that they have hectic schedules, and receiving calls at night is very difficult for them.

*More than me, my wife gets irritated by the phone call. – Clinical expert*

*I have a hectic schedule in the district hospital and receiving nighttime calls is not always possible for me. I will have duty schedule next morning also, and nighttime calls at 1 or 2 am disturb my sleep. – Clinical expert*

### Challenges Faced by Service Providers

Service providers reported 2 major challenges: 1) calls not being readily answered by clinical experts, and 2) phone network problems. Some service providers said that when trying to contact experts, the phone calls were not answered and experts did not call back. They felt that experts were busy and, therefore, they did not try contacting the experts again. A few service providers also mentioned problems with the phone network, which had prevented them from contacting experts.

### Recommendations From Clinical Experts and Service Providers

Most of the clinical experts suggested ensuring availability of essential drugs and equipment to make this program more effective. Several of the service providers and clinical experts reported that video consultation was helpful and should be added in the program. Several of the experts suggested that incentives need to be provided for clinical experts to improve motivation to participate in the consultation service.

### Reasons for Not Using Helpline

Among the service providers who did not use the helpline, the reasons given during the interviews were:

- The presence of an easily accessible nearby referral center (reported by 5 providers)
- The absence of complicated cases at their facility (reported by 6 providers)
- Not knowing about the program (reported by 6 providers)

Among these providers, 2 said they missed the training and 3 were recently transferred from another location. One provider reported calling a nurse at the local district hospital who was not included in our pool of experts.

## DISCUSSION

The MNH helpline program piloted in response to the COVID-19 pandemic to support service providers at PHC facilities of Nepal was found to have been well received among both service providers and clinical experts. The service providers and clinical experts reported that the program was helpful in providing support for making clinical decisions during obstetric emergencies, building confidence and skills of service providers, improving timely referral, and saving patients time and money. Although the program was started as a pilot project during the COVID-19 emergency with plans to implement it for only 1 year, it was found useful even after the pandemic, especially during the monsoon season when travel is difficult.

The helpline program supported service providers in both managing cases inside the health facility and timely referral for more complex cases. The program helped to avoid delays in making referral decisions as well as in reaching the proper referral center by improving the network between peripheral-level health facilities and referral centers. Through this helpline program, we gave the service providers not only mobile phone numbers of experts with whom they could seek support but also a contact list of phone numbers for ambulance/transport, referral centers/hospitals, and local municipal representatives who could support referrals including airlifting, which also helped to avoid delays in arranging transportation. Furthermore, the program team oriented the service providers on airlifting and referral procedures, which included filling referral slips, arranging transport, and informing the referral center about the referred case. Thus, unnecessary delays in referring from one health facility to another health facility were also avoided through proper communication with the referral center. Proper orientation and support in the airlifting process has also helped in reducing administrative delays. Although there was a referral system even before introducing the helpline program, this program has helped to strengthen the referral system and improve its functionality.

Based on these findings, the helpline program was scaled up after the COVID-19 pandemic in other working districts of the organization, especially in Karnali province of Nepal where road access is difficult. The same modality has been continued with greater focus on consulting with clinical experts from within the same district. Providing the printed poster with contacts and mobile credits to service providers in birthing centers is being continued in those districts. Some of the municipalities have also continued this program from their own budget even after the implementing organization has phased out from the district. Health facilities are also better able to airlift the patients on time, showing sustainability of the program.

The study found that the use of MNH helpline was highest during the first few months of the program and slowed down over the next few months but again increased in May and June of 2021. This could be attributed to 3 reasons. First, the use at the start of the program (July and August 2020) was higher due to service provider curiosity about the program and, second, because it coincided with the initial pandemic lockdown and the monsoon season, during which muddy roads are often blocked. Subsequently, the lockdown was relaxed and travel restrictions were lifted and, as a result, use of the MNH helpline also dropped. In May and June 2021, some travel restrictions were imposed again and it was also the rainy season; the use of the helpline increased correspondingly. This implies that the MNH helpline is particularly useful when there are transportation problems to reach a higher-level referral center, requiring providers to reach out for support that they typically would not “need” during normal contexts as they could simply refer patients experiencing complications. Studies have shown that the use of telehealth increased during the COVID-19 pandemic with travel restrictions compared with the pre-pandemic period.^15^ The third reason for the overall decrease in use of the helpline over time could be due to less responsiveness from the clinical experts (i.e., not answering their calls), as described by some of the service providers.

The study also showed that despite the support of clinical experts, various health system challenges hinder adequate service provision—notably lack of essential drugs and medicine and limited knowledge and skills of service providers to manage complications—that cannot be addressed by this program. To make such a service more effective, in addition to providing remote consultation support, it is important to assure the supply of essential drugs and to build clinical competency and confidence of service providers. In Nepal, shortages of essential drugs and medicines are common at PHC facilities. The 2021 Nepal Health Facility Survey has shown that only 6% of health facilities offering vaginal delivery have all 8 essential medicines for mothers.^16^ Thus, concrete efforts are needed from the local government to address this issue. To build clinical competency and confidence of service providers, telehealth can be combined with an education program for more promising results. A study conducted in Latin America that combined telehealth services with virtual trainings, onsite coaching using simulation, and a quality improvement plan decreased perinatal mortality by 29%, the need for blood transfusion by 7%, and eclampsia by 5.5%.^12^ Similarly, another study showed that a model based on education and remote assistance for managing obstetric emergencies reduced the number of referrals due to obstetric emergencies by 65% and also decreased the severity of maternal disease.^17^

### Limitations

One major limitation of this study was the underreporting of cases for which teleconsultations were conducted; service providers did not systematically record all the such cases in their logbooks. This was evident during field visits and field observations by program staff. Clinical experts reported receiving a substantially higher number of calls than recorded in telephone logbooks. The study team was not able to collect data from service providers through direct contact on a regular basis due to limited staff time. Another limitation was the inability to collect baseline data on number of complicated cases and referred cases to enable a before-and-after comparison. These data were not maintained in regular service registers and we did not have time to collect baseline data since the program was designed and implemented during the COVID-19 pandemic. Further, the study would have been interesting to explore the variations in use of the helpline between districts, which is recommended for future study.

### Lessons Learned and Recommendations

Both service providers and the clinical experts felt that it was important to continue this program beyond the acute phase of the COVID-19 pandemic, but sustaining the program imposes challenges since it is based on volunteer work by the clinical experts, several of whom mentioned the need for incentives. It is important to have a dedicated pool of clinical experts willing to provide support 24 hours a day/7 day a week to maintain and sustain such a program. To increase completeness of reporting, control mechanisms such as performance-based payment may be helpful; for example, providing monthly mobile credit balance only after reporting has been completed. In addition, coordination with the referral hospitals to take ownership of this program, with their duty doctors taking responsibility for teleconsultation may help ensure sustainability of this program. A hub and spoke model of teleconsultations can be adapted for sustainability of the program as used in several states of India, where the hub is usually a tertiary-level medical college and the spokes are peripheral-level health facilities.^18^ The feasibility of incorporating video consultations should also be further explored to improve the effectiveness of this program. Further study with a robust pre-post design or a randomized controlled trial is necessary to determine patient-level outcomes due to the teleconsultation program. Also, a costing study or cost-effectiveness study would be beneficial for local level government to implement a similar program in future.

## CONCLUSION

The MNH helpline teleconsultation service was found to helpfully contribute to clinical decision-making of service providers and building their confidence and skills over the acute phase of the COVID-19 pandemic. The helpline helped to increase the percentage of cases successfully managed within the facility, most likely saving lives and reducing costs related to referral and referral delays. With proper modifications, the program has the potential to be scaled up and maintained beyond the COVID-19 era, especially in remote facilities with limited access to higher-level referral centers. Strengthening the pool of dedicated clinical experts by providing incentives, using existing structures, and building strong linkages between PHC facilities and referral centers are essential for sustainability of such a program.

## Notes

**Peer Reviewed**
